# CCR2 Positive Exosome Released by Mesenchymal Stem Cells Suppresses Macrophage Functions and Alleviates Ischemia/Reperfusion-Induced Renal Injury

**DOI:** 10.1155/2016/1240301

**Published:** 2016-10-24

**Authors:** Bing Shen, Jun Liu, Fang Zhang, Yong Wang, Yan Qin, Zhihua Zhou, Jianxin Qiu, Yu Fan

**Affiliations:** ^1^Department of Urology, Shanghai General Hospital, Shanghai Jiaotong University, School of Medicine, 100 Haining Road, Shanghai 200080, China; ^2^Department of Nephrology, Shanghai General Hospital, Shanghai Jiaotong University, School of Medicine, 100 Haining Road, Shanghai 200080, China

## Abstract

Mesenchymal stem cells (MSCs) derived exosomes have been shown to have protective effects on the kidney in ischemia/reperfusion-induced renal injury. However, the key components in the exosomes and their potential mechanisms for the kidney protective effects are not well understood. In our current study, we focused on the abundant proteins in exosomes derived from MSCs (MSC-exo) and found that the C-C motif chemokine receptor-2 (CCR2) was expressed on MSC-exo with a high ability to bind to its ligand CCL2. We also proved that CCR2 high-expressed MSC-exo could reduce the concentration of free CCL2 and suppress its functions to recruit or activate macrophage. Further, knockdown of CCR2 expression on the MSC-exo greatly abolished these effects. Finally, we also found that CCR2 knockdown impaired the protective effects of MSC-exo for the renal ischemia/reperfusion injury in mouse. The results indicate that CCR2 expressed on MSC-exo may play a key role in inflammation regulation and renal injury repair by acting as a decoy to suppress CCL2 activity. Our study may cast new light on understanding the functions of the MSC-exo and these receptor proteins expressed on exosomes.

## 1. Introduction

Renal I/R (I/R) injury can be caused by renal transplantation, leading mainly to acute renal injury (AKI) [1]. Despite the state-of-the-art advanced pharmaceutical therapy, AKI remains one of the major causes of morbidity and mortality in patients hospitalized for renal transplantation [2]. Although various attempts have been made to prevent or treat AKI, most of these efforts have yielded limited success [3–5]. AKI is still a great threat for patients with acute renal I/R injury. Therefore, there is a compelling need to find a new and safe therapeutic method for patients with acute renal I/R injury. Macrophage-mediated inflammation is an invariable finding in acute renal I/R injury [6] and is a critical initial and aggravating factor in renal damage [7]. Renal I/R always rapidly elicits a vigorous inflammatory reaction, inflammatory cell recruitment, cytokine production, and generation of free radicals and oxidative stress [8, 9]. In turn, these factors directly participate in further tissue damage after I/R injury [10, 11]. Therefore, inhibition of the inflammatory reaction is suggested to be pivotal for protecting the kidneys from acute I/R injury.

Previous studies have shown that mesenchymal stem cells (MSCs) from different sources, including human cord blood, bone marrow, embryo, and fetal membranes can be applied in tissue repair, such as promoting recovery from AKI induced by various causes. As it has been reviewed by Dr. Camussi's research group, the numerous registered clinical trials have proposed the use of MSCs to help kidney recovery in patients with AKI or with delayed graft function following kidney transplantation [12]. MSCs not only induce angiogenesis to improve ischemia-related organ dysfunction but also have the capacity of anti-inflammation and immunomodulation for attenuating I/R-induced tissue dysfunction [13–15]. Recent studies have demonstrated that MSCs suppress inflammation through the paracrine mechanism and that microvesicles (MVs) derived from MSCs play a major role in this mechanism [16–18]. Among the various types of MVs, exosomes, which are nanosized extracellular vesicles (30–100 nm in diameter) and are positive for CD9, CD63, and CD81, have been intensely studied recently [19, 20]. Exosomes can mediate cell-cell communication under normal and pathological conditions by shuttling proteins, mRNA, and microRNAs [21]. In light of these findings, more and more research has been conducted with the aim of investigating the functions of exosomes. The effect of exosomes on AKI, hepatic injury, and myocardial I/R injury has been demonstrated previously [22–24]. However, the influences of exosomes on the macrophage-related inflammation over the course of acute ischemic renal injury and repair are not well understood.

In the current study, we found the C-C motif chemokine receptors 2 (CCR2) were enriched in the exosomes derived from mouse bone marrow mesenchymal stem cells. And CCR2 was reported to be associated with the recruitment and activation of the peripheral monocytes/macrophages [25]. The aim of this study was to clarify the role of CCR2 expression on MSC-derived exosomes in macrophage function and renal repairing.

## 2. Materials and Methods

### 2.1. Animals and Cells

All of the Balb/c mice were obtained from the Shanghai SLAC Laboratory Animal Co. Ltd. (Shanghai, China). Strain Balb/c bone marrow mouse mesenchymal stem cells were obtained from Cyagen Biosciences Inc. and cultured using Mouse Mesenchymal Stem Cell Growth Medium (MUCMX-90011, Cyagen Biosciences, Guangzhou, China) according to the manufacturer's instructions. The cells were tested positive for CD44, Sca-1 and negative for CD34, CD117 by flow cytometry analysis and negative for bacteria, fungi, and mycoplasma.

Mouse fibroblast cell line NIH3T3 and macrophage cell line RAW264.7 were obtained directly from the ATCC (Type Culture Collection Committee, Chinese Academy of Sciences). These cells were maintained in RPMI medium 1640 (GIBCO) supplemented with 10% fetal bovine serum, 2 mM L-glutamine, and 25 mM HEPES. All the cell lines were in culture continuously for no more than 6 months prior to analysis.

### 2.2. Isolation and Purification of Exosomes

Exosomes were isolated and purified according to previously established methods [21]. Briefly, cells were cultured in the medium containing exosome-free serum for 48 h and the supernatants were collected. Then, the exosomes were separated by ultracentrifugation and then passed through a 0.22 *μ*m filter. Exosome pellets were resuspended in 1 mL of PBS, with protein content measured via BCA absorbance (Thermo Fisher Scientific Inc.) and stored at −80°C until use.

### 2.3. RNA Isolation and Protein Extraction

Total RNA was extracted using Trizol (Thermo Fisher Scientific, Rockford, IL, USA). Total proteins were extracted using a RIPA buffer (Thermo Fisher Scientific) containing protease inhibitors (Sigma-Aldrich, St. Louis, MO). Protein quantification was carried out using a BCA protein assay kit (Thermo Fisher Scientific).

### 2.4. RNA Transfection

The siRNAs cocktail for human CCR2 (si-CCR2) or negative control RNAs (NC RNA) were designed and synthesized by Shanghai GenePharma Co. Ltd. Shanghai, China. Human UMSCs were transfected used Xfect™ siRNA Transfection Reagent (Clotech, USA) according to the manufacturer's instructions. Briefly, cells were grown in 100 mm plates and transfected individually with RNA at a concentration of 250 pmol/plates. RNA was extracted 48 hours after transfection.

### 2.5. qRT-PCR

For the mRNA analysis, first-strand cDNA was generated using oligo dT and random primers, and the Power SYBR Green PCR Master Mix (Thermo Fisher Scientific) was used for real-time PCR in a StepOne Plus System (Thermo Fisher Scientific). Primers were designed using the Primer Express program. All experiments were performed in triplicate.

### 2.6. Western Blot Analysis

The Western blot analysis was performed according to previously established methods [26]. Briefly, cells were collected and lysed in M-PER Mammalian Protein Extraction Reagent (Pierce, Rockford, IL). All samples were normalized according to the protein concentrations and separated in 10% SDS-PAGE gels and then transferred to nitrocellulose filter membranes (Pall Corp., Washington, NY) using the wet transfer blotting system (Bio-Rad, Hercules, CA). The following antibodies were used for Western blotting: anti-CCR1, anti-CCR2 (Santa Cruz Biotechnology), anti-p-p65 (Abcam), and anti-GAPDH (Abcam) as an endogenous control. Goat anti-mouse CCR2 antibody was obtained from Santa Cruz Biotechnology. The gray levels of blots were analyzed using Image J software.

### 2.7. Enzyme-Linked Immunosorbent Assay (ELISA)

The expression levels of chemokine receptors on the exosomes were examined using the ELISA kit (CUSABIO BIOTECH Co., Ltd., Wuhan, China) according to the manufacturer's instructions. In brief, proteins were measured with a standard curve derived from known amounts, as provided by the manufacture. For competitive analysis of CCL2 binding, recombinant mouse CCL2 protein (100 ng per well, Biolegend, San Diego, CA) was incubated with exosomes in different concentrations for 1 hour. And then the exosomes and exosome-free medium were separated by ultracentrifugation. The CCL2 protein levels in the medium or exosomes were then determined by ELISA analysis.

### 2.8. Renal Ischemia/Reperfusion Model and Exosome Administration

The Babl/c mice were anesthetized with diethyl ether and 2% pentobarbital sodium (30 mg/kg). Then the left renal artery and vein were occluded using a vascular clamp for 60 min through a frank incision. At 10 min after removing the clamp, each mouse received a renal capsule injection of exosomes solution (200 *μ*g exosomes diluted in 20 *μ*L PBS) or only 20 *μ*L PBS into its left kidney. Then the frank incision was closed using 4-0 silk sutures in two layers. The sham operation was performed in a similar manner, except for the clamping of the renal vessels and the injection of exosomes or PBS. Then the mice were sacrificed at day 5 after the treatments and the kidneys were collected for pathologic examination.

### 2.9. Renal Histopathology

One portion of the renal tissue was fixed in 10% buffered formalin followed by embedding in paraffin and staining with hematoxylin and eosin. Additional sections were used for immunohistochemical evaluation of F4/80 (Santa Cruz Biotechnology, Santa Cruz, CA) as a marker for activated macrophage infiltration. Each section was counted in 20 randomly selected high-power fields (×400) by light microscopy (Nikon, Tokyo, Japan). The morphologic findings were scored (0–4, 5 levels) according to cast deposition, tubular dilation, tubular degeneration, and tubular necrosis. Two independent observers with no knowledge of the experimental design evaluated each section.

### 2.10. Renal Function Detection

Blood samples were taken from the abdominal aorta or caudal vein to evaluate renal function. Blood urea nitrogen (BUN) and creatinine (Cr) were measured using standard methods and matched reagents by Hitachi 7600 Analyzer (Hitachi, Tokyo, Japan).

### 2.11. Migration Assay

The macrophage cell migration assay was carried out according to previously established methods [27, 28]. Briefly, the Raw264.7 cell suspension (100 *µ*L, 10^6^ cells/mL) was loaded into the upper wells of the transwell chamber (8 *µ*m pore size; Corning, NY, USA) and exosomes plus CCL2 were used as the chemotactic factors in the lower wells. The plate was incubated at 37°C for 16–20 h. Then the cells remaining in the upper chamber were removed, and the membranes were fixed in ethanol and stained using Giemsa stain. The numbers of migrating cells on the membranes were photographed and quantified using an inverted microscope (Nikon, Tokyo, Japan). All assays were repeated independently at least three times.

### 2.12. Statistical Analysis

Data were presented as mean ± SD. Statistical comparisons between the experimental groups were analyzed using ANOVA and Student's *t*-test. *P* < 0.01 was considered to be statistically significant.

## 3. Results

### 3.1. Identification of CCR2 as an Enriched Functional Protein on MSC-Derived Exosomes (MSC-exo)

**Figure 1:**
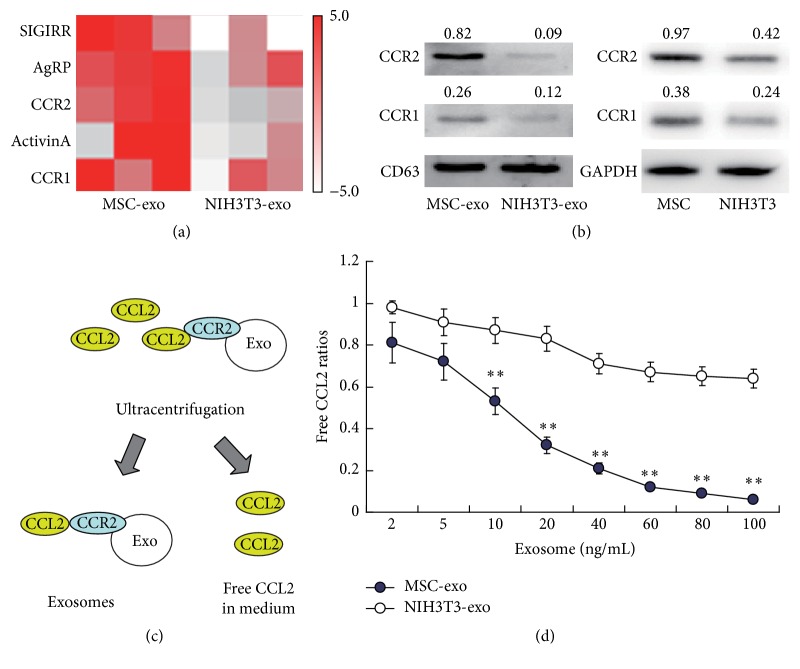
Identification of CCR2 as a key component highly expressed on MSC-derived exosomes (MSC-exo). (a) Screening of specific expressed proteins in bone marrow MSC-derived exosomes (MSC-exo) and NIH3T3 cells-derived exosomes (NIH3T3-exo) by ELISA with three repeat samples in each group. (b) WB analysis of protein expression levels for exosomes and cells. CD63 or GAPDH was used as an internal reference protein for exosomes and cells, respectively. The gray levels of blots were analyzed and the ratios of interesting gene/reference gene were shown. (c)-(d) Competitive analysis for the binding ability of CCR2 positive exosomes. Recombinant mouse CCL2 protein (100 ng per well) was incubated with exosomes in different concentrations for 1 hour. Then these exosomes were removed by ultracentrifugation. The free CCL2 protein levels in the medium were then determined by ELISA analysis and the ratios of free CCL2/total CCL2 were shown (d). Bars represent standard error of the mean ± S.D from three repeats. All the significance was determined by ANOVA and Student's *t*-test. ^*∗∗*^ *P* < 0.01.

We first analyzed the expression levels of more than 40 inflammation-related proteins in the MSC-exos via an ELISA-based scanning using NIH3T3-exos as a control. We found that MSC-exos had a specific protein abundance signature which was quite different from those of the NIH3T3-exos (Figure 1(a)). Among the most differently expressed 5 proteins in the MSC-exos compared to the NIH3T3-exos, we were interested in the two C-C motif chemokine receptor proteins, the C-C motif chemokine receptor-1 (CCR1) and CCR2. These receptor proteins are well known as receptors associated with the recruitment and activation of the peripheral monocytes/macrophages [25]. By WB analysis, we found that both the two receptor proteins were highly expressed in the MSC-exos and MSCs, but the CCR2 was much more abundant in the MSC-exos (Figure 1(b)). And these two proteins were expressed at very low levels in the NIH3T3-exos and the NIH3T3 cells, indicating that these proteins were specifically expressed on the MSC-exos.

To further reveal the possible roles of these receptors, we investigated whether these CCRs expressed on the MSC-exos still had the ability to bind their ligands. We then incubated different concentrations of the MSC-exos and the NIH3T3-exos with the medium containing CCL2, the known ligand of CCR2. The exosomes were then removed using serial ultracentrifugation, and the free CCL2 protein in the exosome-free medium was collected for ELISA analysis (Figure 1(c)). We found that adding the MSC-exos greatly reduced the free CCL2 in the medium according to the concentrations of exosomes, but the NIH3T3-exos had much weaker binding activity (Figure 1(d)). These data indicate that the CCR2 expressed on the MSC-exos was functional and could strongly bind free CCL2 to reduce its concentrations.

### 3.2. CCR2 Knockdown MSC-Derived Exosomes Failed to Bind CCL2

**Figure 2:**
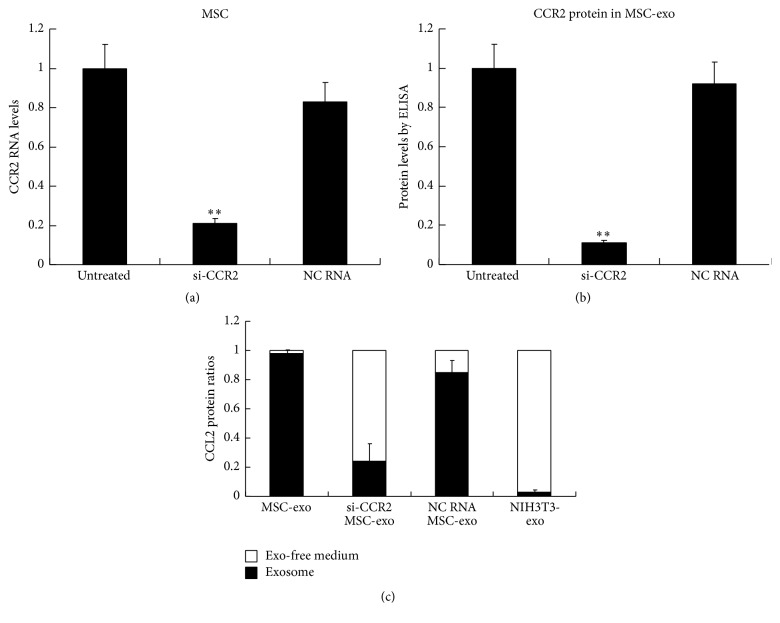
CCR2 knockdown MSC-derived exosomes failed to bind CCL2. (a) CCR2 knockdown efficacy in the MSCs. A CCR2-specific cocktail of siRNAs (si-CCR2) was transfected into the MSCs for 72 hrs, and a scramble RNA was used as a negative control RNA (NC RNA). Quantitative RT-PCR was used to analyze the changes of mRNA levels in these cells and the change folders for each group compared to untreated MSCs were shown. (b) CCR2 expression levels of the exosomes from the conditional mediums of treated MSCs. The CCR2 protein concentrations were determined by ELISA and the change folders for each group compared to untreated MSCs were shown. (c) CCL2 binding efficiency of exosomes. Recombinant mouse CCL2 protein (100 ng per well) was incubated with the exosomes for 1 hour, as indicated by the groups. Then, the exosome-free (exo-free) medium and exosomes were separated by ultracentrifugation. Then, the free CCL2 protein levels in the medium were determined by ELISA analysis and the ratios of free CCL2/total CCL2 were shown. Bars represent standard error of the mean ± S.D from three repeats. All the significance was determined by ANOVA and Student's *t*-test. ^*∗∗*^ *P* < 0.01.

To further validate whether the MSC-exo bound CCL2 through the CCR2 protein, we then tried to establish CCR2 knockdown exosome models. We first transfected CCR2-specific siRNAs into MSCs to knock down the expression levels of CCR2 in the MSCs and then collected the conditional medium of these MSCs for exosome purifications. We found that the siRNAs greatly reduced the CCR2 mRNA levels in MSCs compared to the NC RNA-transfected MSCs (Figure 2(a)). Additionally, the exosomes derived from these MSCs had significantly decreased CCR2 protein expression levels (Figure 2(b)) compared to the control exosomes. We therefore analyzed the CCL2 binding ability of these exosomes using labeled CCL2, with the NIH3T3-exo as a control. We found that the CCR2 knockdown MSC-derived exosomes (si-CCR2 MSC-exos) bound much less of the CCL2 compared to the NC RNA-transfected MSC-derived exosomes (NC RNA MSC-exos) (Figure 2(c)). All of these results demonstrated that the CCL2-binding ability of the MSC-exos was dependent on the CCR2 expression levels, and the si-CCR2 MSC-exos could be used as a model of CCR2 knockdown exosomes in our future studies.

### 3.3. CCR2 Positive MSC-Derived Exosomes Suppress Macrophage Migration and Activation

**Figure 3:**
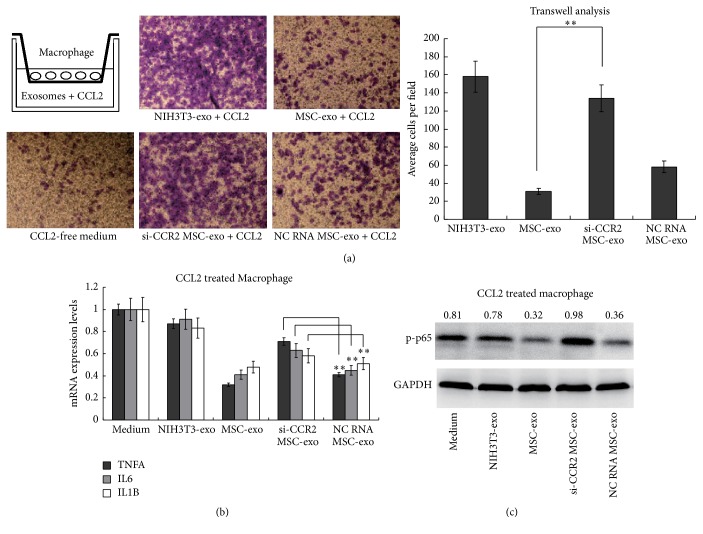
CCR2-positive MSC-derived exosomes suppress macrophage migration and activation. (a) Transwell assay assessing the migration ability of mouse macrophages. The migrated cells on the membranes were counted under the microscope and both the representative figures and averaged cell counts for each group were shown. (b)-(c) Raw264.7 cells were stimulated with CCL2 in the presence of exosomes from the MSCs or NIH3T3 cells. Then, the quantitative RT-PCR analysis was used for the mRNA levels of inflammation factors* TNFA*,* IL6,* and* IL1B* in the macrophages (b). The change folders for each group compared to the CCL2 alone treated macrophages were shown. The WB analysis was used to determine the phosphorylation levels of NF-*κ*B p65 (p-p65) with GAPDH as an internal reference protein. The gray levels of blots were analyzed and the ratios of p-p65/GAPDH were shown (c). Exos: exosomes; si-CCR2 MSC-exo: exosomes derived from CCR2 siRNAs-transfected MSCs; NC RNA MSC-exo: exosomes derived from negative control RNA-transfected MSCs; bars represent standard error of the mean ± SD from three repeats. All the significance was determined by ANOVA and Student's *t*-test. ^*∗∗*^ *P* < 0.01.

Considering the fact that the CCR2 was usually expressed on monocytes and macrophages, promoting their migration and activation in the presence of extracellular CCL2, we therefore wondered whether the CCR2 positive exosomes could regulate mouse macrophage functions. For the in vitro analysis, we firstly established a transwell model to analyze the regulatory functions of the MSC-exo in CCL2-induced macrophage migration. By adding different MSC-exos or NIH3T3-exos into the CCL2-containing medium, we found that MSC-exo greatly suppressed the migration of mouse macrophages compared to the NIH3T3-exo controls. In contrast, si-CCR2 MSC-exos failed to inhibit macrophage migration, whereas NC RNA MSC-exos still had the ability (Figure 3(a)). These results indicated that MSC-exos suppressed the CCL2-induced migration of macrophages, and this effort was greatly dependent on the high-expressed CCR2 protein.

The influence of MSC-exos on CCL2-induced macrophages activation was also investigated. CCL2 was known to induce NF-*κ*B activation and inflammatory factors expression after ligation to the CCR2 in the macrophages. Using qRT-PCR analysis, we found that the MSC-exos and the NC RNA MSC-exos strongly abolished the CCL2-induced elevation of the inflammatory factor mRNAs, such as* TNFA*,* IL6,* and* IL1B*, but the si-CCR2 MSC-exos did not have such effects (Figure 3(b)). By WB analysis, we also found that phosphorylated p65 of NF-*κ*B (p-p65) was significantly decreased in the MSC-exos-treated macrophages compared to that of the NIH3T3-exos group, while si-CCR2 MSC-exos also showed an impaired ability to suppress CCL2-induced NF-*κ*B activation (Figure 3(c)). Therefore, we suggested that CCR2-positive exosomes target CCL2 and suppress its functions to induce macrophages migration and activation.

### 3.4. CCR2-Positive Exosomes Derived from MSCs Reduces I/R-Induced Renal Injury

**Figure 4:**
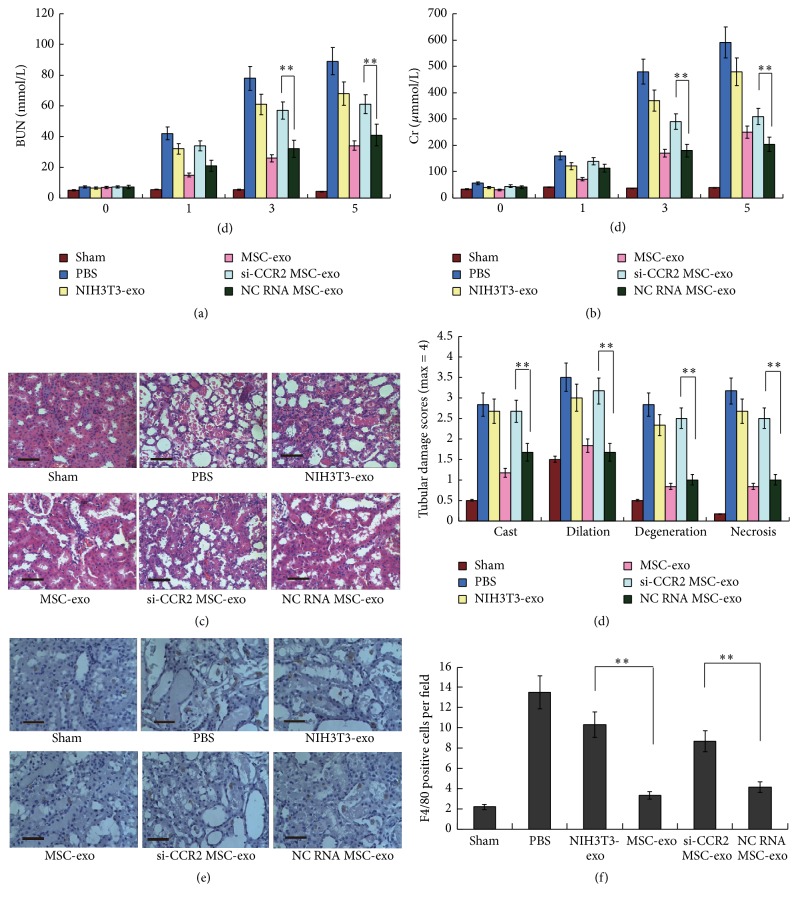
CCR2-positive MSC-derived exosomes reduce ischemia/reperfusion-induced renal injury. Babl/c mice were anesthetized and the ischemia/reperfusion operation was performed by clamping the renal vessels for 60 mins. A sham operation was also performed without renal vessels clamping. At 10 min after the surgery, mice received a renal capsule injection of 200 *μ*g of exosomes per kidney or only PBS as a control. (a)-(b) Blood urea nitrogen (BUN) and creatinine (Cr) values from 0 days to 5 days. Bars represent standard error of the mean ± SD from three repeats. (c)-(d) Representative micrographs of renal histology at day 5 were shown (c). Scale bars, 100 *μ*m. Morphologic findings were also scored according to cast deposition (cast), tubular dilation (dilation), tubular degeneration (degeneration), and tubular necrosis (necrosis), and the average scores for each group were shown as mean ± SD from six repeats (d). (e)-(f) Representative micrographs of F4/80 staining of mice kidney section at day 5 were shown (e), scale bars, 50 *μ*m. The counts of positive cells per field were also shown as the mean ± SD from six repeats (f). All the significance was determined by ANOVA and Student's *t*-test. ^*∗∗*^ *P* < 0.01. Exos: exosomes; si-CCR2 MSC-exo: exosomes derived from CCR2 siRNAs-transfected MSCs; NC RNA MSC-exo: exosomes derived from negative control RNA-transfected MSCs.

Previous studies have revealed that CCL2/CCR2-related macrophage activation plays a key role in the I/R-induced renal injury [29, 30]. To clarify the functions of CCR2-positive MSC-exos in vivo, we therefore established an I/R-induced renal injury mouse model and compared the effects of CCR2-positive and CCR2 knockdown MSC-derived exosomes with those of the NIH3T3 cells or NC RNA-transfected MSCs as controls. PBS was also used as a negative control group. At 1 day after I/R surgery, we observed a significant rise in serum blood urea nitrogen (BUN) and creatinine (Cr) levels in the PBS group, and these values increased over time. Renal function was clearly improved from day 3 to day 5 after the rats were treated with the MSC-exos or the NC RNA MSC-exos. However, the BUN and Cr levels in the si-CCR2 MSC-exos and the NIH-3T3-exos groups had no notable changes compared to the PBS group (Figure 4(a)). Lesions were observed at day 5 by H&E staining of kidney tissue slices. The slices showed that numerous necrotic areas of the proximal epithelium had appeared and abundant tubular protein casts had formed in the si-CCR2 MSC-exos, PBS, and NIH-3T3-exos groups; however, the tubular lesions markedly decreased in the MSC-exo and NC RNA MSC-exos groups (Figure 4(b)). We also examined the levels of macrophage infiltration in the kidney of these mice by F4/80 staining on the slices. It was found that both the MSC-exos and the NC RNA MSC-exos treatments decreased the macrophage infiltration after I/R surgery, while the si-CCR2 MSC-exos and the NIH-3T3-exos groups had no notable changes compared to the PBS group (Figure 4(c)). All of these results indicate that the MSC-derived exosomes may promote the recovery of I/R-induced kidney injury through a CCR2-dependent manner.

## 4. Discussion

The renal ischemia and reperfusion have been proved to induce renal microvascular endothelium activation and elevated chemokines expression, which always mediate monocytes adhesion and migration into injured tissue [31]. As one of the most important chemokine contributing to the proinflammatory monocytes recruitment, CCL2 and its major receptor CCR2 have been proved to be key factors that regulate the tubular injury after renal I/R, and recruitment of circulating CCR2+ monocytes into the renal tissue plays a critical role in promoting tubular injury following I/R injury in the studies based on CCR2 knockout mice models [29, 30]. In our current study, CCR2 was found to be enriched in the MSC-derived exosomes, and these CCR2 positive exosomes strongly bound extracellular CCL2 and reduced its concentration. We therefore suggested that the CCR2 positive exosomes played as decoys to suppress CCL2 function and then subsequently inhibited the recruitment and activation of the peripheral monocytes/macrophages. The role of the CCR2 positive exosomes may increase our understanding of the protective effect of exosomes derived from MSCs and may provide a novel target for therapeutic studies.

It has been well known that exosomes may function as a carrier as they are composed of a lipid bilayer and contain proteins and RNAs. Most previous studies have shown that exosomes could mediate cell-cell communications by transferring the proteins or RNAs into target cells [21]. However, although many cell-surface receptors are enriched on various exosomes, the biological functions of these receptors were less discussed. Here we found that exosomal CCR2 proteins could strongly bind the CCL2 and then reduce free CCL2 concentrations. We therefore suggest that the CCR2 positive exosomes may function as endogenous CCL2 sponges to consume these ligands and block their biological functions. Considering the very large amounts of exosomes used in the MSC-exo based therapies, we suggested that this function may be powerful enough to suppress endogenous cytokines and other pathological factors and may play a key role in their mechanism. Our finding may also provide a model to understand the biological functions of exosomal surface receptors in many other fields.

## 5. Conclusions

In this study, we found that a membrane receptor CCR2 was enriched in the MSC-derived exosomes, and these CCR2 positive exosomes strongly bound extracellular CCL2 and reduced its concentration. We therefore suggest that CCR2 positive exosomes played as decoys to suppress CCL2 functions and then subsequently inhibited the recruitment and activation of the peripheral monocytes/macrophages. The role of CCR2 positive exosomes may increase our understanding of the protective effect of exosomes derived from MSCs and may provide a novel target for renal injury therapeutic studies. Our finding may also provide a model to understand the biological functions of exosomal surface receptors in many other fields.
